# Performance of IRBs in China: a survey on IRB employees and researchers’ experiences and perceptions

**DOI:** 10.1186/s12910-022-00826-4

**Published:** 2022-08-29

**Authors:** Xing Liu, Ying Wu, Min Yang, Yang Li, Kaveh Khoshnood, Esther Luo, Lun Li, Xiaomin Wang

**Affiliations:** 1 Center for Clinical Pharmacology, The Third Xiangya Hospital, Central South University, Changsha, 410013 Hunan People’s Republic of China; 2 Medical Ethics Committee, Xiangya Hospital of Central South University, 87 Xiangya Road, Changsha, 410008 Hunan People’s Republic of China; 3 School of Literature and Journalism, Central South University, Changsha, 410012 Hunan People’s Republic of China; 4 Xiangya School of Nursing, Central South University, Changsha, 410013 Hunan People’s Republic of China; 5 Shanghai Topgen Bio-Pharm Co, Ltd, Shanghai, 200135 People’s Republic of China; 6 Yale School of Public Health, Yale University, 60 College Street, New Haven, CT 06520 USA; 7 Faculty of Humanities and Social Sciences, Dalian University of Technology, Dalian, 116023 Liaoning People’s Republic of China

**Keywords:** China, Institutional review board, Performance

## Abstract

**Background:**

Performance evaluation is vital for IRB operations. As the number of IRBs and their responsibilities in reviewing and supervising clinical research grow in China, there is a significant need to evaluate their performances. To date, little research has examined IRB performance within China. The aim of this study was to ascertain the perspectives and experiences of IRB employees and researchers to (1) understand the current status of IRBs; (2) compare collected results with those of other countries; and (3) identify shortcomings to improve IRB performance.

**Methods:**

This study was conducted in China from October 2020 to September 2021, using an online survey with the IRB-researcher assessment tool-Chinese version.

**Results:**

757 respondents were included in the analysis and classified into IRB employees, researchers, or those who are both IRB employees and researchers. Overall, the score for an ideal IRB was significantly higher than that of an actual IRB. Compared to the US National Validation study, Chinese participants and American participants both agree and differ in their perspectives on the most and least important ideal items.

**Conclusion:**

This investigation provides a benchmark of the perceived performance of actual IRBs in China. IRBs in China can be precisely adjusted by targeting identified areas of weakness to improve their performances.

## Background

All research (i.e., biomedical, behavioral, social science, and epidemiological research) that involves human subjects should be submitted to an institutional review board (IRB) for periodic review, including prior to conducting a study [1]. An IRB is an independent body that operates to ensure the protection of the rights, safety, and well-being of human subjects [2]. While the functions of IRBs around the world and across institutions may differ slightly, their general responsibilities in ethical review and procedures are the same [3, 4]. In general, ethical reviews are legally mandated and must be obtained ahead of the conduct of human subjects research [5].

Performance evaluation can be an effective tool in identifying areas of vulnerability in an IRB to subsequently improve its quality and efficiency [6]. Evaluating the performance of an IRB though remains a challenging task [7]. Perspectives from IRB members and researchers are fundamental then to understanding IRB performance, and several studies have targeted this concept [8]. To date, IRB performance has been examined in more than 14 countries worldwide such as in Europe and the Americas [9–15], Africa [16–22], and Asia [23–26]. One study conducted in low to middle-income countries (LMICs) focused on IRB chairpersons to evaluate whether the IRB’s functions complied with recognized international standards [27]. Another study in Myanmar chose IRB representatives to assess the structures and processes of Research Ethics Committees (RECs) at medical institutions [24]. Investigators in Jordan evaluated the awareness and attitudes of healthcare investigators toward the structure and importance of IRBs in that region [28]. Research conducted in Singapore targeted biomedical researchers and support staff to gain a general understanding of the perceptions surrounding IRB functions and characteristics [29]. Lastly, recent research in China has indicated that IRBs routinely face issues related to performance, including the absence of supervision, unclear review criteria, the limited competency of ethics committees (including inadequate knowledge of ethics), and poor follow-up of reviews [30].

Before 2019, China’s IRBs operated and conducted ethical reviews based on regulations and guiding documents issued by the National Health Commission (NHC) and National Medical Product Administration (NMPA)—formerly the China Food and Drug Administration (CFDA) [31, 32]. Since 2019, IRBs in China have been confirmed with legislative statuses, resulting in the strict enforcement of ethical reviews of clinical research. Subsequently, IRBs have gradually gained their legal status through various laws, such as The Civil Code which states that whenever clinical studies are necessary for the development of new drugs, medical devices, or new prevention and treatment methods, they should be approved by the IRB [33]. Additionally, according to the Chinese Biosecurity Law, clinical research on new biomedical technologies should also be approved by ethical review [34]. The Law of the People’s Republic of China on Basic Medical and Health Care and Health Promotion requires clinical trials of drugs and medical devices and other medical research to be conducted in accordance with standards in medical ethics and approved by ethical reviews [35]. China’s Drug Administration Law further requires that clinical trials of drugs be implemented in accordance with ethical principles, with study proposals formulated for examination and approval by an IRB [36]. Finally, the Physician Law of the People’s Republic of China states that physicians who carry out clinical trials of drugs and medical devices and other types of clinical studies must abide by medical ethics and seek approval by an IRB following the law [37].

In 2018 and 2019, NMPA and NHC changed previous qualifications for the certification of clinical trials for medical devices and drugs, respectively, into a registration policy. Specifically, the basic condition that institutions conducting clinical trials should meet for registration is having an IRB [38, 39]. As a result, many IRBs were established in newly registered research institutes. As of February 2, 2022, 1,214 and 1,108 institutes in drug and medical device trials, respectively, have been registered [40]. Performance evaluations can standardize and strengthen IRBs and their review processes [41]. Therefore, it is imperative to evaluate IRBs, so that IRBs can be improved based on empirical data. Our study included IRB employees (including IRB members and staff) and investigators since IRB employees and researchers have the most direct perspectives on IRB performance, making this study distinct from past research. For instance, Keith-Spiegel et al. sampled researchers almost exclusively [42], while Markus K. Labude et al. investigated Singaporean biomedical researchers/research support staff [29]. Jonathan C. Reeser’s study population included research coordinators, IRB members, as well as investigators [43]. Daniel E. Hall’s study population consisted of principal investigators/project coordinators and IRB members/staff [44]. While Tiffany Chenneville’s study in India included IRB members and faculty investigators [45], similar to our study, her study population mainly drew from medical colleges, whereas our participants came from both medical and health institutions, with a small number from medical schools. Due to differences between studies, including the separate aims and purposes, it can be difficult to make a horizontal comparison. However, the general perspectives on IRBs’ performance can help to understand and distinguish the gaps between different countries [44].

Currently, there are no empirical studies in China evaluating these groups’ perspectives and experiences within IRBs. Our study aims to use the IRB-Researcher Assessment Tool Chinese Version (IRB-RAT-CV) to investigate IRB employees and researchers in China. In addition to understanding perspectives on the characteristics and functions of IRBs, this study also facilitates the comparison of these findings with studies conducted in other countries, so as to comprehend identified differences and improve IRB operations.

## Methods

### Study design

From October 2020 to September 2021, a national cross-sectional study was conducted to assess the performance of IRBs in China. Participants were recruited using WeChat through convenience sampling. In China, residents primarily communicate through this free messaging and calling app. It is the most popular communication app in China and has approximately 549 million active users per month [46]. For this study, we targeted WeChat groups, meaning conversations between more than two users sharing several common characteristics or goals; in this case, we aimed for WeChat groups consisting of IRB members and researchers [47]. Every year, China holds nationwide academic exchange and training activities for IRBs and researchers. IRB staff and researchers from different regions of the country participate in the training and establish WeChat groups from these activities. Thus, several groups exist on WeChat that consist of IRB staff and researchers with different professional backgrounds from various regions of China. An online questionnaire, accessible through a link, was distributed to these WeChat groups. This method allowed us to recruit IRB-related staff and researchers from different areas across China.

The questionnaire was divided into three parts: informed consent, a basic demographic sheet, and the IRB-RAT-CV. The first part included the informed consent form, which described the purpose as well as the risks and benefits of the study. If participants clicked “Agree”, the web page would directly jump to the next portion with the demographic sheet and IRB-RAT-CV questionnaire.

The participants of this study were IRB employees and researchers from medical colleges and hospitals nationwide, with IRB employees including chairpersons, vice chairs, members, and staff, and researchers including those who have undertaken at least one biomedical research project involving human participants. Participants could designate their role as one or more of the positions mentioned above, and selections were categorized into either “IRB Employee”, “Researcher” or both. As a result, three roles were created for analysis: IRB employee, researcher, and those who are both an IRB employee and researcher. Participation in the survey was voluntary and anonymous.

### Sampling method

The Cochrane formula (*n* = z2pq/e2) was used for the sample size calculation, with a confidence interval of 0.95 and a margin of error of 5%. The resulting minimal sample size was 385. Considering the questionnaire may be completed incorrectly or there may be a significant amount of missing data, the total number of questionnaires was increased by 20%. Therefore, the final sample size of the study was estimated to be 462. A total of 757 respondents were ultimately included in our study.

### The IRB-RAT-CV instrument

The instrument IRB-RAT has been used in the United States [42, 43], Singapore [29], India [45], and Peru [48]. It is a self-reported measure of IRB performance that consists of 45 items describing a variety of IRB activities and functions [42]. The questionnaire assesses the relative importance of these items to participants across eight themes. The eight themes include: procedural justice (how the decision-making process is carried out); absence of bias (a feature of procedural justice); pro-science sensitivity and commitment; interactional justice (interpersonal sensitivity and justification); formalities (an IRB’s formal functioning, structure, and composition); upholding the rights of human research participants; IRB outreach (offering services beyond those mandated); and competence (how competently the IRB performs its functions) [42]. Respondents in this study were asked to give two Likert Scale ratings on each item, to indicate both the importance of that item within their conception of an ideal IRB and how closely the item describes the actual IRB they work within. In our study, an ideal IRB is understood as an IRB that displays features that are most central in enabling the study participants to achieve their best work while an actual IRB is understood as relating to the IRB features those participants feel their IRB displays. Specifically, the survey asks, “As an investigator, how important is each item to you in your work? First, rate, along a seven-point continuum, how important each item would be to you in performing your best work, with 7 = ‘Absolutely essential’ to 1 = ‘Not important’. Next, for the same item, along another seven-point scale, rate how well that item describes your actual IRB, with 7 = ‘Highly descriptive’ to 1 = ‘Not at all descriptive.’” [31].

The reliability and validity of the IRB-RAT-CV (See the Appendix for summaries of the 45 items). were confirmed previously by our research team [31]. The Cronbach’s alpha coefficients for the ideal IRB and actual IRB were 0.989 and 0.992, while the Spearman-Brown coefficients were 0.964 and 0.968, respectively. Item-total correlation values ranged from 0.631 to 0.886, and 0.743 to 0.910. Confirmatory factor analysis for the ideal IRB yielded χ2/df = 2.811 l; root mean square error of approximation (RMSEA) = 0.062; normed fit index (NFI) = 0.904; Tucker-Lewis Index (TLI) = 0.931; and comparative fit index (CFI) = 0.936. Confirmatory factor analysis for the actual IRB yielded χ2/df = 2.967; root mean square error of approximation (RMSEA) = 0.065; normed fit index (NFI) = 0.914; Tucker-Lewis Index (TLI) = 0.936; and comparative fit index (CFI) = 0.941. This is consistent with values indicating adequate reliability and validity.

### Data analysis

Analysis was conducted using SPSS 26.0 (IBM, Chicago, IL, USA). Both descriptive and inferential statistics were used. The categorical variables are described in terms of frequency and percentage, and the median of the inter-quartile range (IQR) was used for the continuous variable. Nonparametric Mann–Whitney U test and the Kruskal–Wallis (KW) test were used to assess the association between actual and ideal scores with the characteristics of respondents, with p values less than or equal to 0.05 considered statistically significant.

## Results

### Participant characteristics

A total of 757 respondents were included in the analysis, of which 44.6% were IRB employees, 50.5% were researchers, and 4.9% were both IRB employees and researchers. The demographic characteristics of the participants are shown in Table 1. Among the three age groups, most participants were over 41 years old, and the majority (62.1%) were female. The proportion of those with a master’s degree (43.3%) was higher than those with a bachelor’s degree and below (28.0%) and those with a Ph.D degree (28.7%). Most participants (84.3%) reported having received ethics training in the previous three years. Most participants (86.5%) also worked in hospitals.

**Table 1:** Characteristics of participants

Characteristics (*n* = 757)	Number (*n*)	Percentage (%)
*Participants*		
IRB employee	338	44.6
Researcher	382	50.5
Both IRB employee and researcher	37	4.9
*Gender*		
Male	287	37.9
Female	470	62.1
*Studied abroad for more than 3 months*		
Yes	155	20.5
No	602	79.5
*Received ethics training in the last 3 years*		
Yes	638	84.3
No	119	15.7
*Age (years)*		
18–30	104	13.8
31–40	325	42.9
≥ 41	328	43.3
*Experience (years)*		
0–5	349	46.1
6–10	237	31.3
11–20	129	17
≥ 21	42	5.6
*Professional title*		
Primary level professional title	83	11
Intermediate level professional title	241	31.8
Associate professor	221	29.2
Professor	146	19.3
None	66	8.7
*Level of education*		
Ph.D	217	28.7
Master’s degrees	328	43.3
Bachelor's degree or below	212	28.0
*The IRB where you work has an independent ethics committee office*		
Yes	589	77.8
No	111	14.7
Do not know	57	7.5
*Institution*		
Tertiary (level 3) and secondary (level 2) hospital	655	86.5
Medical school	66	8.7
Others (e.g. Life medicine research institute, law office, colleges, community and centers for disease control and prevention)	36	4.8

IRB = institutional review board

### The effect of different characteristics on the overall ideal score and actual score

Table 2 compares the effects of participants’ characteristics on scoring. In general, participants’ rating for an ideal IRB was significantly higher than that for their actual IRB. Participants who received ethics training in the last three years had higher scores for their ideal IRB and actual IRB than those who did not receive ethics training in the last three years (*P* < 0.05). Participants with an independent IRB office at their IRB had statistically significant differences in ratings with greater requirements for the ideal IRB and satisfaction with actual IRB services compared to those with no independent office (*P* < 0.05). Similarly, participants in tertiary and secondary hospitals rated actual IRBs higher than participants in medical schools (*P* < 0.05). There were no statistically significant associations between ideal and actual scores and other demographic variables (role, sex, age, study abroad experience, length of employment, job title, and education) (see Table 2).

**Table 2:** The effect of demographic factors on ideal IRB and actual IRB

Variable	Median (IQR)		*P-*value*	
	Ideal IRB	Actual IRB	Ideal IRB	Actual IRB
*Participants*				
IRB employee	6 (6–7)	6 (5–6)	0.564	0.292
Researcher	6 (6–7)	6 (5–6.5)		
Both IRB employee and researcher	6 (6–7)	6 (5–6)		
*Gender*				
Male	6 (6–7)	6 (5–7)	0.52	0.115
Female	6 (6–7)	6 (5–6)		
*Studied abroad for more than 3 months*				
Yes	6 (6–7)	6 (5–6.5)	0.281	0.15
No	6 (6–7)	6 (5–6)		
*Received ethics training in the last 3 years*				
Yes	6 (6–7)	6 (5–6.5)	0.003	0.006
No	6 (6–7)	6 (5–6)		
*Age (years)*				
18–30	6 (6–7)	6 (5–6)	0.369	0.911
31–40	6 (6–7)	6 (5–6)		
≥ 41	6 (6–7)	6 (5–6)		
*The IRB where you work has an independent ethics committee office*				
Yes	6 (6–7)	6 (5–7)	< 0.0001	< 0.0001
No	6 (6–7)	5 (5–6)		
Do not know	6 (5–6)	6 (5–6)		
Yes vs No			0.657	0.04
Yes vs Do not know			< 0.0001	< 0.0001
No vs Do not know			0.022	0.232
*Experience (years)*				
0–5	6 (6–7)	6 (5–7)	0.406	0.594
6–10	6 (6–7)	6 (5–6)		
11–20	6 (6–7)	6 (5–6)		
≥ 21	6 (6–7)	6 (6–6)		
*Professional title*				
Primary level professional title	6 (6–7)	6 (5–6)	0.661	0.087
Intermediate level professional title	6 (6–7)	6 (5–7)		
Associate professor	6 (6–7)	6 (5–6)		
Professor	6 (6–7)	6 (5–6)		
None	6 (6–7)	6 (5–6)		
*Level of education*				
Ph.D	6 (6–7)	6 (5–6)	0.649	0.668
Master’s degrees	6 (6–7)	6 (5–6.5)		
Bachelor’s degree and below	6 (6–7)	6 (5–6)		
*Institution*				
Tertiary (level 3) and secondary (level 2) hospital	6 (6–7)	6 (5–6)	0.341	0.006
Medical school	6 (6–7)	5.5 (5–6)		
Others	6 (6–7)	6 (5–7)		
Tertiary (level 3) and secondary (level 2) hospital vs Medical school				0.004
Tertiary (level 3) and secondary (level 2) hospital vs others				1.00
Medical school vs others				0.193

*Mann-Whitney U Test and Kruskal-Wallis Test were used

*IRB*, institutional review board; *IQR*, Interquartile range

### Perspectives of different roles within IRBs on the most important and least important ideal items

In addition to highlighting the ideal items which respondents in our study regarded as the most and least important, Table 3 compares the perspectives of the Chinese sample with the US National Validation (USNV) sample since the author Keith-Spiegel et al. originally developed the IRB-RAT and used it as a tool to evaluate IRB performance. Overall, Chinese participants and American participants seem to both agree and differ on which ideal items are the most and least important (see the Appendix for descriptions of the 45 items). Specifically, researchers in both cohorts agree that an IRB that reviews protocols in a timely fashion (item 1) is one of the most important items, falling within the top 5. In particular, among the 45 items, American researchers considered item 1 (6.43 ± 0.80) as the most important ideal item [42]. In terms of the ranking of the least important ideal features, Chinese and American researchers nearly hold the same opinions, in perceiving an IRB offering consultation during the development of protocols and applications, an IRB providing editorial suggestions for documents and protocols, an IRB having a diverse membership, and an IRB being composed of more than one public member (item 32, item 34, item 40, and item 41, respectively) as the least important.

**Table 3:** Comparison of top-ranking ideal items between the Chinese sample and USNV sample by mean scores

Chinese sample						USNV sample	
IRB employee		Researcher		Both IRB employee and researcher		Researcher	
Item number	Mean ± SD	Item number	Mean ± SD	Item number	Mean ± SD	Item number	Mean ± SD
*Top 5 most important ideal characteristics*							
(42)	6.58 ± 0.69	(43)	6.30 ± 0.79	(15)	6.46 ± 0.89	(1)	6.43 ± 0.80
(15)	6.57 ± 0.70	(44)	6.30 ± 0.79	(35)	6.46 ± 0.76	(13)	6.17 ± 1.10
(44)	6.56 ± 0.70	(15)	6.29 ± 0.78	(3)	6.43 ± 0.75	(18)	6.10 ± 1.11
(3)	6.47 ± 0.71	(36)	6.28 ± 0.75	(42)	6.43 ± 0.75	(19)	6.08 ± 1.19
(30)	6.47 ± 0.75	(1)	6.28 ± 0.85	(1)	6.41 ± 0.91	(23)	6.01 ± 1.16
*Top 5 least important ideal characteristics*							
(45)	5.85 ± 1.35	(41)	5.90 ± 1.05	(9)	5.89 ± 1.03	(41)	2.68 ± 1.69
(29)	5.86 ± 1.25	(34)	6.08 ± 0.88	(39)	5.92 ± 1.12	(34)	3.20 ± 1.82
(9)	5.95 ± 1.04	(45)	6.09 ± 0.93	(38)	5.92 ± 1.05	(33)	4.03 ± 1.68
(32)	6.01 ± 1.03	(40)	6.10 ± 0.90	(29)	5.92 ± 0.91	(40)	4.07 ± 1.93
(34)	6.02 ± 1.06	(32)	6.13 ± 0.81	(45)	5.95 ± 1.09	(32)	4.30 ± 1.76

Related summarized item content
(1) Reviews protocols in a timely fashion
(3) Provides complete rationale for changes regarding protocols
(9) Works with investigators to resolve disagreements
(13) Members do not allow personal biases to affect their work
(15) Requires members to abstain from a review when there is a conflict-of-interest
(18) Upholds participants’ rights while facilitating the conduct of research
(19) Does not use its power to suppress research and avoid criticism
(23) Members are knowledgeable about IRB procedures and national policy
(29) Composed of members regarded as competent investigators
(30) Provides a training program for new members
(32) Offers consultation during the development of protocols and applications
(33) Offers opportunities to educate investigators about national research policy
(34) Offers editorial suggestions for documents and protocols
(35) Members understand and act within the scope of their functions
(36) Maintains accurate records
(38) Requires chair to be an experienced investigator
(39) Monitors approved research projects according to national policy
(40) Has a diverse membership
(41) Composed of more than one public member
(42) Primary function is to protect human participants
(43) Takes appropriate action when there is scientific misconduct
(44) Acts promptly when its decisions are violated by an investigator
(45) Applies flexible standards for informed consent requirements

*USNV*, US national validation; *SD*, Standard deviation; *IRB*, institutional review board

Within the Chinese sample, participants with different roles in the IRB also ranked ideal items differently. The three roles (IRB employee, researcher, and those who are both an IRB employee and researcher) all agreed that IRB members should be required to abstain from a review when there are conflicts-of-interest (item 15), and such protocols are essential to the development of IRBs, ranking this item among their top 5 most important ideal items. Specifically, those who are both IRB employees and researchers ranked item 15 as the most important (6.46 ± 0.89). On the other hand, IRB employees and researchers regarded an IRB’s primary function as protecting human participants (item 42) (6.58 ± 0.69) and an IRB that takes appropriate action when there is scientific misconduct (item 43) (6.30 ± 0.79), respectively, as the most important item. All three roles also agreed that an IRB that applies flexible standards for informed consent requirements (item 45) belongs as one of the least important ideal items, of which, IRB employees considered this item to be the least important.

### The difference in participants’ ratings for each theme

Table 4 shows the mean scores of actual IRB and ideal IRB across eight themes. In terms of the actual overall mean score, researchers (5.80 ± 0.91) scored the highest, and for the ideal overall mean score, IRB employees (6.27 ± 0.61) scored the highest. In evaluating the actual IRB, scores for researchers on procedural justice, interactional justice, IRB competence, IRB outreach, IRB formal functioning, structure, and composition, and upholding the rights of human research participants were higher than the other two groups. Regarding the absence of bias and pro-science sensitivity, IRB employees scored higher than the other groups.

**Table 4:** Comparison, by role, of mean factor domain scores awarded to actual IRB and the ideal IRB

Domain	IRB employee	Researcher	Both IRB employee and researcher
Procedural justice	5.71 ± 0.95	5.79 ± 0.92	5.63 ± 0.89
	6.34 ± 0.63	6.23 ± 0.69	6.28 ± 0.75
Interactional justice	5.76 ± 0.91	5.77 ± 0.94	5.64 ± 0.84
	6.20 ± 0.70	6.20 ± 0.69	6.12 ± 0.76
Absence of bias	5.89 ± 0.88	5.83 ± 0.93	5.76 ± 0.94
	6.36 ± 0.67	6.26 ± 0.71	6.28 ± 0.70
Pro-science sensitivity	5.82 ± 0.90	5.81 ± 0.94	5.72 ± 0.76
	6.26 ± 0.71	6.22 ± 0.73	6.20 ± 0.66
IRB competence	5.62 ± 0.97	5.79 ± 0.97	5.60 ± 0.76
	6.25 ± 0.68	6.22 ± 0.70	6.18 ± 0.69
IRB outreach	5.58 ± 1.03	5.75 ± 1.01	5.51 ± 0.61
	6.09 ± 0.85	6.13 ± 0.77	6.04 ± 0.76
IRB formal functioning, structure, and composition	5.76 ± 0.96	5.79 ± 0.94	5.78 ± 0.85
	6.27 ± 0.70	6.15 ± 0.73	6.18 ± 0.74
Upholding the rights of human participants	5.83 ± 0.99	5.85 ± 0.96	5.72 ± 0.99
	6.36 ± 0.68	6.24 ± 0.73	6.25 ± 0.68
Total	5.75 ± 0.86	5.80 ± 0.91	5.67 ± 0.76
	6.27 ± 0.61	6.21 ± 0.67	6.20 ± 0.67

Data represents mean ± standard deviation; *IRB*, institutional review board

In terms of the scores for the ideal IRB, IRB employees provided higher scores on procedural justice, absence of bias, pro-science sensitivity, IRB competence, IRB formal functioning, structure, and composition, and upholding the rights of human research participants compared to the other two groups. Researchers meanwhile scored higher on IRB outreach than the other two groups.

## Discussion

In our study, both roles (IRB employee and researcher) had higher ideal means than actual means on 45 items, and this difference was statistically significant (*P* < 0.05). Those who are both IRB employees and researchers had higher ideal means than actual means on 44 items (except for item 41, an IRB that is composed of more than one public member, in which the ideal mean is equal to the actual mean), and the difference was statistically significant (*P* < 0.05). This indicates that those who are both IRB employees and researchers believe that no improvements are necessary for IRB membership, as their actual IRBs are most likely composed of multiple public members.

In addition, the highest and lowest rated items in our sample differed from those in the USNV sample. The distinct cultures between China and the US may have resulted in these differences in participants’ values and ethical consciousness [45]. Compared with the USNV sample’s score for an ideal IRB (5.19 ± 1.50), Chinese researchers (6.21 ± 0.67) had higher expectations for their IRBs. This is perhaps due to how Chinese IRBs operate largely under policies set at the national level in China in recent years, so the demands placed on IRBs are considerable and increasing in this region.

### Participant characteristics and their effect on ideal and actual scores

In terms of the characteristics of the participants in our study, 43.3% of respondents were over 41 years old, and the majority (62.1%) were female; this is consistent with how females in general make up 65% of IRBs in China. Contrastingly, Chenneville’s study revealed that 73% and 83% of IRB members at the two IRBs of interest, respectively, were male [45]. On the surface, these percentages may seem high, but they are likely consistent with the male-dominated ethics committees in India. It is interesting to note that Chenneville’s figures also mimic the typical gender distribution of IRBs in the US. In a systematic review of the empirical literature evaluating IRBs in the US, Abbott and Grady found that the composition was predominantly male [49].

Most participants (77.8%) reported that the IRB they worked for had an independent IRB office to coordinate administrative tasks which indicates that the development of IRBs in China is becoming professionalized and specialized. A majority of participants (84.3%) also reported having received ethics training in the previous three years. This finding demonstrates that participants have a high enthusiasm to engage in ethical training, which is conducive to the cultivation and promotion of the subjects’ ethical awareness in their work. Such a finding can be significant for IRBs in China, as some studies have shown that training even a small number of individuals can change policies and practices in research ethics [50]. In addition, most participants (86.5%) worked in hospitals, reflecting how hospitals in China serve as both medical and health institutions, with established IRBs.

### The influence of participants’ characteristics on the overall ideal and actual scoring

In our study, the ideal score exceeded the actual score across all eight themes, indicating that participants’ ideal IRB functioning was greater than actual IRB functions. With these data, IRBs in China can be precisely adjusted according to the IRB-RAT-CV results to improve areas with low scores, thus improving the quality of IRBs [45]. As the discrepancies between actual and desired IRB functions are likely to be common, they can be utilized as positive indicators for high ideals that may contribute to interest in seeking significant improvements in IRBs [45]. In general, the score for ideal IRB is significantly higher than that for actual IRB, indicating that participants have higher expectations and requirements for IRBs.

Moreover, the vast majority of participants’ ratings on the ideal performance of an IRB were significantly higher than the USNV sample in Keith-Spiegel et al.’s study (except item 1, an IRB that reviews protocols in a timely fashion) [42] and exceeds the ideal score of the India-based study on most items (30 items) [45]. The ideal score of the Chinese sample was found to be more consistent with that of the Singaporean one [29]. In terms of actual scores, the ones in the Chinese study are higher than the actual scores of the Indian and Singaporean samples across the 45 items, with more than half of the items (23 items) being one point higher.

### Perspectives of different roles on the most important and least important ideal items

The differences between the three roles in their views of the most and least important ideal themes are reflected in the following aspects. Firstly, regarding the most important items, in our study, IRB employees thought that an IRB whose primary function is to protect human participants (item 42) to be the most important ideal theme (6.58 ± 0.69). This is very similar to studies in which researchers in the US and Singapore placed a high value on preserving the rights of participants [29, 42]. In contrast, researchers, and those who are both IRB employees and researchers considered an IRB that reviews protocols in a timely fashion (item 1) as one of the most important ideal items. These results were consistent with the USNV sample [42]. Ranking the timeliness of IRB review processes as an important IRB characteristic reflects researchers’ desire to avoid unnecessary delays in carrying out their studies. Untimely reviews are not simply a matter of inconvenience for researchers; such delays may also have detrimental impacts on interpersonal relationships within IRBs which may consist of IRB members and members of the research community. At present, most protocols in China must undergo ethical review before they can be submitted to the national human genetic resources office, to meet requirements for sample and data collection approval and subsequently be implemented in clinical trials [51]. Based on the ideal items deemed the most important in this study, an IRB can continue to improve in these areas to better its services and IRB members’ and researchers’ satisfaction.

Regarding the least important items, IRB employees, and those who are both IRB employees and researchers thought that an IRB that works with investigators to resolve disagreements and an IRB that is composed of members regarded as competent investigators (item 9 and item 29) belong among the least important ideal items. This may indicate that collaboration between researchers and IRBs is not an important factor in IRB operations for these two groups. In contrast, respondents in the Singapore and USNV studies valued item 9, demonstrating how these regions view such collaborations as essential to the timely approval of their research projects (this item was the 3rd highest ideal score in the Singaporean study and the 10th highest ideal score in the USNV sample) [29].

In addition, researchers in the Chinese and USNV samples seemed to agree on which ideal items were the least important; both samples agreed that an IRB that offers consultation during the development of protocols and applications, an IRB that offers editorial suggestions for documents and protocols, an IRB that has a diverse membership, and an IRB composed of more than one public member (item 32, item 34, item 40, and item 41, respectively) are the least important. Perhaps for these researchers, IRB membership is not as significant and has little relevance to their interests. In regards to item 41, the findings in the Singapore and USNV studies were similar to ours: having diverse IRBs with multiple lay members was considered less important than other items [42]. Researchers in the US may perceive laypersons as being unqualified to evaluate research proposals [52]. Compared to the Singaporean survey, item 34 was also a low-ranked ideal item in our study. Given that IRBs are tasked with evaluating ethical and regulatory aspects of a study, correcting typographical and grammatical errors may seem trivial or irrelevant to their functioning, even though these errors do impact the accuracy, readability, and comprehension of proposals and reports [29]. Similarly, both the Singaporean cohort and the USNV sample viewed lay membership, diversity of membership, as well as editorial suggestions from the IRB as some of the least important features [29].

### The difference in participants’ ratings for each theme

This study sheds light on both researcher and IRB employee perceptions of IRBs in China. Overall, there was little difference between the ideal and actual scores of these two groups, and their views were relatively consistent (*P* > 0.05).

Across the eight themes, the difference between the Chinese sample and the samples from India and Singapore in the ideal score is small, but the actual score of the Chinese sample is higher than that of the other samples. In particular, on procedural justice, interactional justice, pro-science sensitivity, and IRB outreach, the actual score of the Chinese sample was much higher than that of the Indian and Singaporean samples (the difference was greater than one point), indicating that Chinese participants were more satisfied with the services provided by their actual IRBs in these four aspects. Similarly, the ideal score of the Chinese sample was significantly higher than that of the USNV sample in all eight themes especially in three themes (IRB competence, IRB outreach, and IRB formal functioning, structure, and composition), with the difference being greater than one point.

## Conclusions

To our knowledge, this is the first study evaluating IRB performance in China, in a general manner using the IRB-RAT-CV tool. Overall, the experiences and perceptions of IRB employees and researchers as well as specific strengths and weaknesses of IRBs in China were identified. In addition, the IRB-RAT-CV facilitated comparisons with evaluations conducted in other countries and pinpointed areas for improvement within Chinese IRBs. There are at least three key insights that can be gleaned from our study.

First, the IRB-RAT-CV demonstrated Chinese IRBs’ strong performances in several areas, including respecting investigators (item 10), maintaining accurate records (item 36), having diverse memberships (item 40), and preserving the protection of human participants as its primary function (item 42). However, the importance of these items differed across the three roles in our study. For instance, an IRB whose primary function is to protect human participants (item 42) was among the most important ideal characteristics for IRB employees and those who are both IRB employees and researchers but not for researchers, whereas, an IRB that has diverse membership (item 40) was one of the least important for researchers but not for the other two groups. Understanding these differences, gleaned from IRBs’ current performances as well as members’ and researchers’ perceptions, is critical in constructing and sustaining infrastructures that support and protect research participants.

Utilizing the perspectives of the three roles in the study, to identify differences between the ideal and actual scores, can highlight to IRBs and institutions areas in which more time and effort should be devoted to improving IRB performance. In particular, our results revealed that IRBs in China can improve in the following items: reviewing protocols in a timely manner (item 1), conducting a comprehensive review of protocols (item 2), seeking outside assistance (item 7), having members who are knowledgeable about IRB procedures and national policy (item 23), providing training programs for new members (item 30), and having sufficient resources to carry out IRB functions (item 37). Items 1 and 30 were among the top 5 most important ideal characteristics for either IRB employees, researchers, or those who are both IRB employees and researchers, emphasizing the significance of these aspects to these participants and in strengthening IRBs.

Lastly, by comparing our results with other IRB evaluations, we can learn from various countries’ experiences to improve aspects in which IRBs in China are lagging. As an example, in our study, all three roles were aware of the importance of IRB members in avoiding conflicts of interest (COI) (item 15), with this item being listed among the top 5 for two of our studied roles but was not among the most important for the USNV sample. Despite these differences in awareness and significance, there are currently no specific regulations for COIs in clinical research in China [32]. Thus, we suggest the following recommendation by Gregor Scherzinger and Monika Bobbert, to be adopted in IRBs across China: IRBs should maintain an up-to-date, publicly available registry in which members can declare any relationships that may lead to conflicts of interest; in addition, IRBs should include rules that specify when members should withdraw from ethics review [53]. Comprehending similarities and differences, as well as the contexts in which they occur, between IRBs across regions can not only identify areas for improvement but also allow China to draw from empirical data as well as other countries’ experiences to strengthen IRB performance.

One limitation of this study was that the inclusion criteria for our sample were limited to IRB employees and researchers. However, other staff and individuals involved in research and IRB operations may have distinct, valuable experiences and perceptions toward IRBs in China. As these views were not included in the present study, the current comparisons and recommendations may be limited. Lastly, because the questionnaire was completed by participants who were more familiar with the operations of an IRB, IRB performance may have been overestimated. Although convenience sampling can result in an overestimation or underestimation of the studied outcome, this may not be a significant limitation in our study because a large proportion of adults in China use WeChat.

In conclusion, we gained a general understanding of China’s current IRB situation, with the collected data providing a benchmark of the perceived performance of actual IRBs in China. Moreover, the data were expanded upon by comparing them with those of other countries, to highlight differences between IRBs in various regions. These results serve to identify areas for improvement and provide empirical data and important references which may be useful in guiding institutions as well as in aiding China to formulate policies in IRB evaluation.

## Data Availability

The datasets used and/or analyzed during the current study are available from the corresponding author on reasonable request.

## Appendix: IRB-RAT 45 item content, with descriptions and summaries.

**Table:**

IRB-RAT item	Summarized item
1. An IRB that reviews protocols in a timely fashion	Reviews protocols in a timely fashion
2. An IRB that conducts a conscientious and complete review of protocols	Comprehensively reviews protocols
3. An IRB that gives a complete rationale for any required changes to or disapprovals of protocols	Provides complete rationale for changes regarding protocols
4. An IRB that includes a complete rationale when it denies or mandates changes in a protocol based on criteria that are more stringent than or different from national research policy	Provides complete rationale for decisions based on stringent criteria
5. An IRB that is open to reversing its earlier decisions	Open to reversing earlier decisions
6. An IRB that invites investigators to present their position whenever a question or concern about a research protocol arises	Invites investigators to present their positions when questions arise
7. An IRB that recognizes when it lacks sufficient expertise to evaluate a protocol and seeks outside experts	Recognizes when it lacks expertise and seeks outside assistance
8. An IRB that responds in a timely manner to investigators’ inquiries about its processes and decisions	Responds promptly to investigators’ inquiries
9. An IRB that works with investigators to find mutually satisfying solutions whenever disagreements exist	Works with investigators to resolve disagreements
10. An IRB that treats investigators with respect	Treats investigators with respect
11. An IRB that acknowledges full responsibility for its errors or delays in processing protocols and attempts to correct them as expeditiously as possible	Takes full responsibility and action in resolving errors
12. An IRB that is open and pleasant in its interactions with investigators	Pleasantly interacts with investigators
13. An IRB with members who do not allow personal biases to affect their evaluation of protocols	Members do not allow personal biases to affect their work
14. An IRB whose members hold no preconceived biases against particular research topics	Members hold no biases towards particular research topics
15. An IRB that requires members to abstain from evaluating protocols whenever a real or apparent conflict-of interest arises	Requires members to abstain from a review when there is conflict-of-interest
16. An IRB whose members hold no preconceived biases against particular research techniques	Members hold no biases towards particular research techniques
17. An IRB that is open to innovative approaches to conducting research	Open to innovations in research
18. An IRB that does a good job of upholding participants’ rights while, at the same time, facilitating the conduct of research	Upholds participants’ rights while facilitating the conduct of research
19. An IRB that does not use its power to suppress research that is otherwise methodologically sound and in compliance with national policy whenever it perceives potential criticism from outside the scientific community	Does not use its power to suppress research and avoid criticism
20. An IRB that views itself as an investigator’s ally rather than as a hurdle to clear	Views itself as ally to investigators
21. An IRB that shows considerable evidence that the advancement of science is part of its mission	Demonstrates that its mission is to advance science
22. An IRB that shows empathy with the difficulties that can present themselves during the design and conduct of research	Shows empathy over the difficulties present in research
23. An IRB with members who are very knowledgeable about IRB procedures and national policy	Members are knowledgeable about IRB procedures and national policy
24. An IRB that conducts a conscientious analysis of potential benefits weighed against potential risks before making decisions	Weighs potential benefits against risks before coming to a decision
25. An IRB that can competently distinguish exempt from nonexempt research	Can distinguish exempt from nonexempt research
26. An IRB that ensures that at least one member is knowledgeable about the content domain of submitted protocols	At least one member knows the content of submitted proposals
27. An IRB whose members arrive at meetings well-prepared	Members come to meetings well-prepared
28. An IRB with a Research Compliance Officer (or staff member in charge of IRB functions) who has a research background	Has Research Compliance Officer with research background
29. An IRB that is composed primarily of members regarded as highly competent investigators	Composed of members regarded as competent investigators
30. An IRB that provides a comprehensive training program for its new members	Provides a training program for new members
31. An IRB that offers information to improve the chances of gaining IRB approval	Offers information to improve chances of approval
32. An IRB that offers consultation during the development of research protocols and grant applications	Offers consultation during the development of protocols and applications
33. An IRB that offers investigators opportunities to be educated about national research policy	Offers opportunities to educate investigators about national research policy
34. An IRB that offers editorial suggestions regarding consent documents and protocols	Offers editorial suggestions for documents and protocols
35. An IRB whose members fully understand and act within the scope of their function	Members understand and act within the scope of their functions
36. An IRB that maintains accurate records	Maintains accurate records
37. An IRB that is allocated sufficient resources to carry out its functions	Has sufficient resources to carry out its functions
38. An IRB that requires that its Chair be an experienced investigator	Requires Chair to be an experienced investigator
39. An IRB that monitors the progress of each approved research project in line with national policy	Monitors approved research projects according to national policy
40. An IRB that has a diverse membership (i.e., includes women, minorities and junior and senior members of the institution)	Has a diverse membership
41. An IRB that is composed of more than one public member	Composed of more than one public member
42. An IRB that views protection of human participants as its primary function	Primary function is to protect human participants
43. An IRB that takes timely and appropriate action whenever scientific misconduct is alleged	Takes appropriate action when there is scientific misconduct
44. An IRB that takes timely action when an investigator has violated its decisions	Acts promptly when its decisions are violated by an investigator
45. An IRB that applies appropriately flexible standards regarding voluntary and informed consent requirements (e.g., required wording is not as demanding for minimal risk research as it is for more risky research)	Applies flexible standards for informed consent requirements

## Abbreviations

IRB: Institutional review board
IRB-RAT-CV: Institutional review board-researcher assessment tool- Chinese version
LMIC: Low to middle-income country
REC: Research ethics committees
NHC: National health commission
NMPA: National medical product administration
CFDA: China food and drug administration
RMSEA: Root mean square error of approximation
NFI: Normed fit index
TLI: Tucker Lewis index
CFI: Comparative fit index
SPSS: Statistical product and service solutions
IQR: Inter-quartile range
KW: Kruskal–Wallis
USNV: US national validation
